# Antioxidant Potential of Lactoferrin and Its Protective Effect on Health: An Overview

**DOI:** 10.3390/ijms26010125

**Published:** 2024-12-26

**Authors:** Quintín Rascón-Cruz, Tania Samanta Siqueiros-Cendón, Luis Ignacio Siañez-Estrada, Celina María Villaseñor-Rivera, Lidia Esmeralda Ángel-Lerma, Joel Arturo Olivas-Espino, Dyada Blanca León-Flores, Edward Alexander Espinoza-Sánchez, Sigifredo Arévalo-Gallegos, Blanca Flor Iglesias-Figueroa

**Affiliations:** Laboratorio de Biotecnología I, Facultad de Ciencias Químicas, Universidad Autónoma de Chihuahua, Circuito Universitarios s/n Nuevo Campus Universitario, Chihuahua 31125, Mexico; qrascon@uach.mx (Q.R.-C.); tsiqueiros@uach.mx (T.S.S.-C.); lsianez@uach.mx (L.I.S.-E.); p336080@uach.mx (C.M.V.-R.); esme.angel.lerma@gmail.com (L.E.Á.-L.); p337882@uach.mx (J.A.O.-E.); p375694@uach.mx (D.B.L.-F.); eaespinoza@uach.mx (E.A.E.-S.); sareval@uach.mx (S.A.-G.)

**Keywords:** lactoferrin, antioxidant, neuroprotector effect

## Abstract

Chronic diseases, including cardiovascular and neurodegenerative diseases and cancer, are significant global health challenges. Oxidative stress, characterized by an imbalance between reactive oxygen species (ROS) production and antioxidant defenses, is a critical factor in the progression of these pathologies. Lactoferrin (Lf), a multifunctional iron-binding glycoprotein, has emerged as a promising therapeutic agent due to its potent antioxidant, anti-inflammatory, and iron-regulating properties. Lf plays a pivotal role in iron homeostasis by chelating iron, modulating its cellular uptake, and reducing ROS production, thereby mitigating oxidative stress-related tissue damage. Lf also demonstrates neuroprotective potential in diseases like Parkinson’s and Alzheimer’s, where it alleviates oxidative damage, regulates iron metabolism, and enhances antioxidant defenses. Furthermore, its ability to enhance endogenous antioxidant mechanisms, such as superoxide dismutase and glutathione peroxidase, underscores its systemic protective effects. Lf’s anti-inflammatory and antimicrobial activities also contribute to its broad-spectrum protective role in chronic diseases. This review consolidates evidence of Lf’s mechanisms in mitigating oxidative stress and highlights its therapeutic potential as a versatile molecule for preventing and managing chronic conditions linked to oxidative damage.

## 1. Introduction

**Figure 1:**
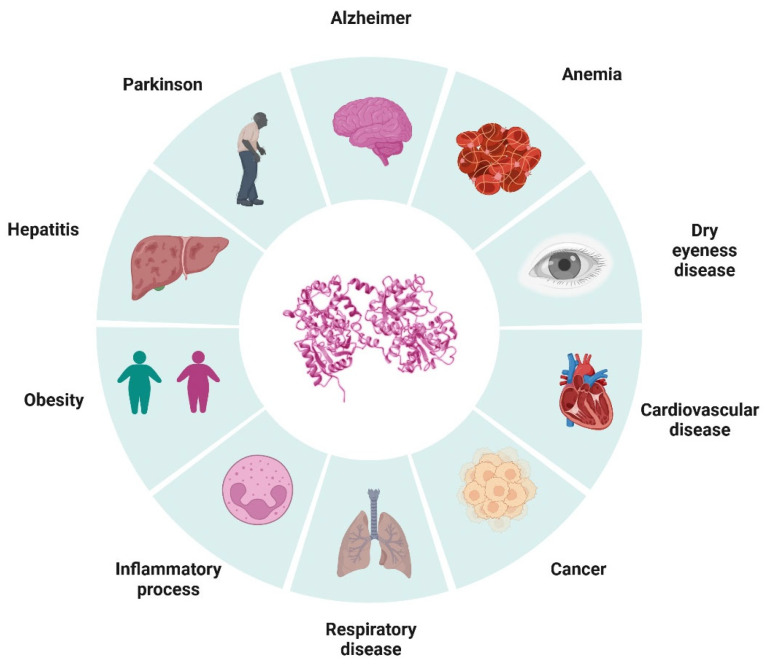
Pathological processes and diseases in which lactoferrin could exert a protective effect through its antioxidant activity (created in https://BioRender.com). Lactoferrin, through its antioxidant capacity, could contribute to the neutralization of reactive oxygen species (ROS) and to cell protection. This mechanism suggests its potential effect in modulating oxidative damage and preventing the development of various chronic pathologies.

Chronic diseases represent a significant public health burden worldwide, with a devastating impact on the quality of life of millions of people [1,2]. Among these diseases, cardiovascular diseases, neurodegenerative diseases, and cancer stand out, which constitute some of the leading causes of morbidity and mortality globally [3,4,5]. It has been observed that the development and progression of these pathologies are closely related to oxidative stress, an imbalance between the production of reactive oxygen species and the body’s antioxidant capacity to neutralize them [6,7]. In this context, antioxidants emerge as a promising strategy to mitigate the detrimental effects of oxidative stress and potentially prevent or slow the progression of these diseases [8]. In this review, we explore the antioxidant role of lactoferrin, an important molecule of the immune system, and its implications in protecting against chronic diseases, focusing on its potential to counteract pathological mechanisms associated with cardiovascular and neurodegenerative diseases and cancer, among others (Figure 1).

## 2. Lactoferrin Generalities and Structure

**Figure 2:**
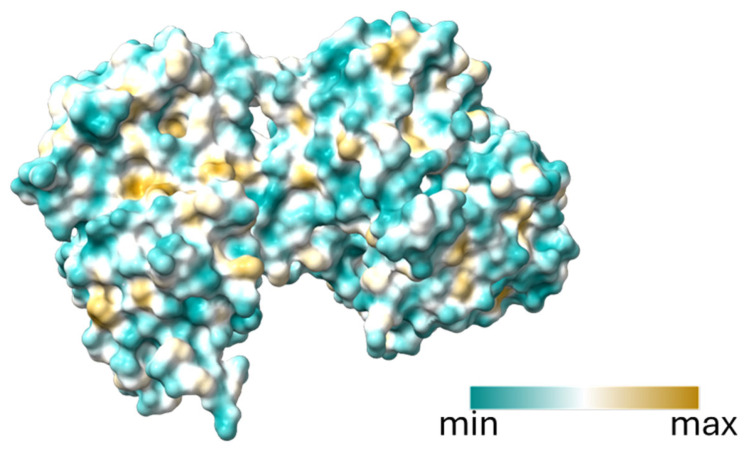
Lactoferrin hydrophobicity profile. The figure highlights regions of hydrophobic (yellow) and hydrophilic (blue) surfaces on the lactoferrin molecule, emphasizing areas critical for receptor binding and molecular interactions. Minimum, −28.89; mean, −6.123; maximum, 22.83.

Lactoferrin (Lf) constitutes a non-heme iron-binding glycoprotein within the transferrin family that is widely distributed throughout the body [9]. It is produced mainly in milk and colostrum (1 g/L and 7 g/L, respectively) and is secreted in practically all body fluids [10]. A fraction called serum lactoferrin is present in the secondary granules of neutrophils, which, in response to inflammatory stimuli, degranulate, thus releasing lactoferrin into the bloodstream, which can reach a concentration of 200 μg/mL [11]. Lactoferrin has an approximate molecular weight of 80 kDa and comprises a single polypeptide chain of about 700 amino acids, with certain variations depending on the species [12]. Its structure is organized into two similar globular lobes, known as N-terminal (amino acids 1–222) and C-terminal (amino acids 344–703), joined by a hinge region containing an alpha helix structure between amino acids 333–343, which gives flexibility to the molecule [13]. These lobes present a combination of alpha helix structures and beta-folded sheets, forming two domains for each of these lobes. Each lobe is capable of binding Fe^2+^ or Fe^3+^ atoms in cooperation with the carbonate ion. Also, it has been reported that Lf can bind to Cu^2+,^ Zn^2+^, and Mn^2+^ ions [14]. On the other hand, an important characteristic of lactoferrin, which may impact some of its functions, is its glycosylated structure. Human Lf has three potential N-glycosylation sites, while bovine Lf has five potential N-glycosylation sites; therefore, glycan diversity is considered one of the factors underlying the differences in the biological properties of lactoferrins [15]. The glycans in the lactoferrin show high binding affinity to a diverse array of cell receptors, in either immune cells or other cells. A family of lectins, including the scavenger receptor C-type lectin (SR-CL) on endothelial cells, DC-SIGN on antigen-presenting cells, hepatic lectin 1 on hepatocytes, Siglec-1 (CD169) on macrophages, and Toll-like receptor 4 (TLR-4) on immune cells, recognizes the glycosidic fraction of lactoferrin (bovine or human) between these receptors. These interactions enable lactoferrin to play key roles in modulating inflammation and defending against pathogens [16,17]. On the other hand, several receptors interact with lactoferrin domains, including intelectin-1 on enterocytes and eosinophils, as well as nucleolin located on the surface of dividing cells. In addition, lactoferrin binds to the low-density lipoprotein-related receptor (LRP1) on monocytes. Other relevant molecular targets include CD14, CD206, and CXCR4, which play key roles in processes such as regulation of the immune system and cell migration [18]. Binding of lactoferrin to these receptors not only facilitates its endocytosis but also activates several signaling pathways, such as mitogen-activated protein kinase (MAPK), which plays a critical role in cell proliferation, differentiation, and survival. These interactions are fundamental for the function of the innate and adaptive immune systems, neuroprotection, and iron metabolism, highlighting the versatility and biological relevance of lactoferrin in multiple physiological processes [16]. Another property that influences lactoferrin’s interactions with receptors is its hydrophobicity (Figure 2), which affects the interaction of the immune system and the nervous system. Such interactions are vital for their role in modulating immune responses and promoting cell signaling [19].

## 3. Lf Implication on Iron Metabolism

**Figure 3:**
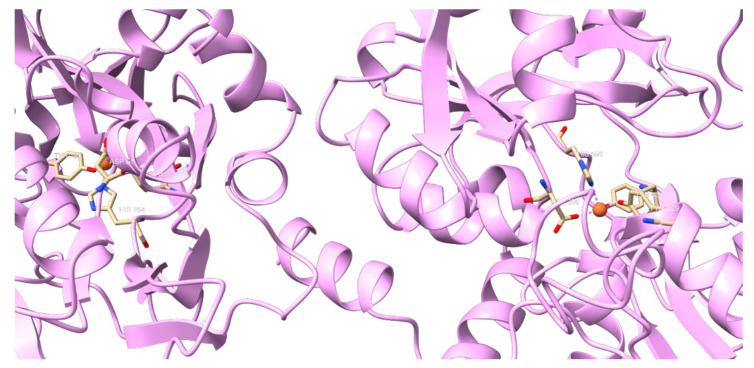
Lactoferrin binds two Fe^3+^ ions. In orange are Fe^3+^ ions; beige residues bind Fe^3+^.

Iron is a vital element required for all living organisms and participates in a wide range of metabolic activities, including oxygen transport, energy metabolism, DNA synthesis, and detoxification [20]. Iron is the most abundant trace element in the human body and can be found as ferric (Fe^3+^) and ferrous (Fe^2+^) ions [21]. The daily diet provides 10% of the iron body, while the remaining 90% is recycled iron, especially from the senescent red blood cells [22]. The Fe^3+^ ion is the most stable form of iron, while the Fe^2+^ is more unstable; in addition, Fe^2+^ is more reactive due to its additional electron, which increases its tendency to generate reactive oxygen species (ROS); thereby, free iron must be strictly regulated [23]. Iron is regulated at both systemic and cellular levels; hepcidin is the main protein in systemic regulation and inhibits iron absorption by blocking ferroportin. Ferroportin is responsible for transporting intracellular iron into the bloodstream in cells that store or absorb iron, such as hepatocytes, macrophages, or enterocytes. On the other hand, iron is stored in cytosol primarily in the form of ferritin, a protein complex that safely sequesters iron. Finally, once in the bloodstream, it is safely transported by transferrin (Tf) to the bone marrow or into those cells that demand iron [22,23,24] As a member of the transferrin family, Lf can reversibly bind two Fe^3+^ ions (Figure 3). However, Lf has a higher affinity for iron than Tf, allowing it to retain iron even under conditions of low pH 3.5. This gives it an advantage over Tf, as it provides to be functional under adverse conditions. Therefore, Lf plays a greater role in regulating absorption in the intestine [25,26]. The main site of absorption is the duodenum; Fe^3+^ is reduced to Fe^2+^ by duodenal cytochrome b (DCYTB) to be internalized by the apical surface of enterocytes. Iron is transported into cells by divalent metal transporter 1 (DMT1) [27]. However, Lf-carrying Fe^3+^ needs a specific receptor (LfR) discovered in the intestinal epithelium’s brush border membranes [28]. The LfR pathway endocytoses iron from Lf; iron release involves a reverse process of iron binding with a structural change, the presence of LfR, and the reduction of Fe^3+^ to Fe^2+^ to be efficiently absorbed [27]. Outside the cell, Fe^2+^ needs to be oxidized to Fe^3+^ in order to be transported; Lf can bind to the ferroxidase ceruloplasmin, which allows the simultaneous conversion of Fe^2+^ to Fe^3+^, while Fe^3+^ binds to Lf [22]. Returning to the fact that iron is a potential producer of free radicals and ROS, the iron chelation by Lf keeps it in a nonreactive state, reducing the availability of Fe^2+^ to produce free radicals. This is important to prevent tissue damage, which can generate chronic diseases such as Alzheimer’s [28]. The following sections will provide a detailed description of this mechanism.

Another way to modulate iron is the regulation of ferroptosis. In a recent study, Lf inhibited ferroptosis in alcohol-induced liver injury (AALI), where this type of death contributes to the development of the injury. Lf reduced the expression of the transferrin receptor and divalent metal transporter 1 while it upregulated ferritin and ferroportin proteins, resulting in a decrease in ferroptosis [29]. However, Lf has a dual effect in ferroptosis; in cancer, Lf may help to upregulate ferroptosis depending on its iron saturation [30].

Finally, it is well known that Lf has antimicrobial activity [12]; one of its mechanisms of action is iron sequestration [31]; iron chelation reduces the availability of iron in the environment. By reducing the availability of iron, Lf creates a hostile environment for microorganisms since it is an essential element for the growth and survival of pathogens; moreover, iron is necessary for the formation of biofilms; bovine Lf, in a concentration of a 100 μg/mL, inhibits biofilm formation of some bacteria such as *Escherichia coli* [32].

Lactoferrin (Lf) plays a critical role in iron metabolism and offers multiple protective functions in the body. Its main activities related to iron include iron sequestration, protection against pathogens, modulation of ferroptosis, and facilitation of iron uptake. Together, these mechanisms contribute to maintaining iron homeostasis in the body, which is crucial for preventing both iron deficiency and overload conditions [33,34].

## 4. Lf Effect in Oxidative Stress

Mitochondria represent the main endogenous source of reactive oxygen species (ROS) production due to their role in ATP synthesis through oxidative phosphorylation. During this process, molecular oxygen (O_2_) is reduced to water (H_2_O) in the electron transport chain. In particular, superoxide generation in mitochondria constitutes a significant source of ROS in cells. The controlled production of free radicals is essential for proper cellular functioning, providing defense mechanisms and promoting survival within physiological ranges. However, an imbalance between the production and neutralization of ROS can lead to the accumulation of reactive intermediates that are potentially harmful, thus contributing to the development of oxidative stress [35].

**Figure 4:**
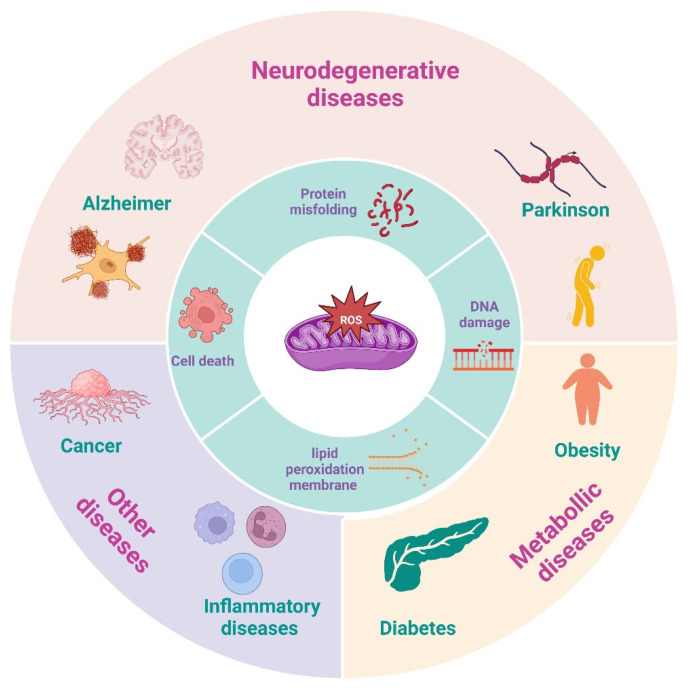
ROS-mediated cellular damage and its implications in important chronic diseases. Although the presence of reactive oxygen species (ROS) is essential for regulating homeostasis in the body, their excessive accumulation can initially trigger damage to organelles and macromolecules, compromising cellular integrity. In later stages, this imbalance can cause significant alterations that contribute to the development of various pathologies such as neurodegenerative, metabolic, and inflammatory diseases and cancer (created in https://BioRender.com).

Oxidative stress arises from an imbalance between the production of reactive oxygen species (ROS) and the body’s ability to neutralize their harmful effects. At the cellular level, the impacts of oxidative stress can manifest in various alterations, including lipid peroxidation in membranes, protein misfolding or processing, and the induction of DNA mutations [36,37]. These molecular modifications, in turn, are associated with the development of various diseases (Figure 4), including neurodegenerative disorders, cardiovascular diseases, cancer, inflammatory diseases, and metabolic syndrome [38,39]. Lactoferrin has demonstrated significant potential in mitigating oxidative stress in various biological systems [40].

### 4.1. Reduction of Reactive Oxygen Species (ROS) Levels

Lactoferrin has been shown to directly reduce intracellular levels of reactive oxygen species. Pre-treatment with lactoferrin significantly decreased ROS production in human mesenchymal stem cells (hMSCs) exposed to hydrogen peroxide (H_2_O_2_). This reduction helps protect cells from oxidative damage induced by excessive ROS, suggesting that lactoferrin can maintain cellular viability and function under oxidative stress conditions [41]. Additionally, bovine lactoferrin has been shown to lower elevated plasma hydrogen peroxide levels associated with oxidative stress-induced conditions, such as dexamethasone-induced hypertension in rats, when it was administrated at a dosage of 400 mg/kg bw/day, indicating its systemic antioxidant effect [42]. Moreover, it was demonstrated that lactoferrin supplementation effectively mitigated mercury-induced oxidative stress in male Wistar rats [42]. Mercury is a potent inducer of ROS, and the cited study highlighted lactoferrin’s role in reducing mercury-induced lipid peroxidation and improving antioxidant enzyme activities, such as superoxide dismutase (SOD) and glutathione peroxidase (GPx). These findings underscore lactoferrin’s potential in reducing oxidative damage caused by heavy metal exposure.

### 4.2. Enhancement of Antioxidant Defense Mechanisms

Lactoferrin not only directly neutralizes ROS but also enhances the body’s endogenous antioxidant defenses; in a dexamethasone-induced hypertension model, it has been observed that oral administration of lactoferrin in a dose-dependent effect significantly boosted the ferric reducing antioxidant power (FRAP) in plasma, indicating an overall improvement in the body’s antioxidant capacity [43]. This enhancement of the body’s antioxidant defense system suggests that lactoferrin can serve as a critical component in managing oxidative stress-related hypertension. Moreover, it has been demonstrated that lactoferrin helps regulate iron homeostasis, which is crucial in preventing iron-induced oxidative stress. By binding free iron, lactoferrin reduces its availability for participation in the Fenton reaction, a process that generates highly reactive hydroxyl radicals [22]. Finally, lactoferrin’s ability to regulate iron metabolism is essential to its antioxidant effects, particularly in scenarios where iron imbalance is present. By controlling iron availability, lactoferrin mitigates oxidative stress, helping to maintain cellular health in conditions involving iron dysregulation [44]. It has been reported that the mechanism by which lactoferrin manages to enter cells is through clathrin-mediated endocytosis; all forms of lactoferrin bind to the lactoferrin receptor. This receptor is expressed in various cells and tissues, such as the small intestine, liver, bones, brain, platelets, and lymphocytes [45]. Lactoferrin also binds to or interacts with receptors other than LfR [46]. These receptors include CD14, cytokine receptor 4, and Toll-like receptor 4 to mediate immune responses [47,48,49], including the role of low-density lipoprotein receptor-related protein 1 in the transport of lactoferrin, the functions of fibroblasts, and the survival of osteoblasts [50,51]. Lactoferrin exhibits a high degree of versatility, as it can interact with a variety of molecules, including heparan sulfate proteoglycans, metal ions, glycosaminoglycans, lipopolysaccharides, and DNA. This multifunctional nature of lactoferrin contributes to its diverse biological roles [52].

Ginet et al. proposed that lactoferrin (Lf) could exert beneficial effects on inflammatory brain injury associated with prematurity. In this study, Lf was supplemented along with maternal feeding during the lactation period, while lipopolysaccharide (LPS) was administered into the subcortical white matter of rat pups on postnatal day 3 (P3). The results demonstrated that Lf attenuates both acute and long-term brain alterations induced by LPS, supporting its potential as a preventive and neuroprotective approach in neonatal encephalopathy associated with prematurity. The article highlights that Lf can stimulate the expression of key antioxidant enzymes, such as superoxide dismutase (SOD), catalase (CAT), and glutathione peroxidase (GPx), while also reducing lipid peroxidation mediated by malondialdehyde (MDA). Additionally, it documents its ability to interact with specific cellular receptors (LfR), activating signaling pathways that promote the expression of genes involved in antioxidant defense, such as those regulated by the transcription factor Nrf2 (nuclear factor erythroid 2-related factor 2) [53]. In a study conducted by Mulder and colleagues, the researchers examined the immunomodulatory properties and antioxidant potential of an oral bovine lactoferrin supplement in humans. They observed statistically significant increases in T cell activation (mediated by CD3+), activation of T-helper cells (mediated by CD4+), and cytotoxic T cell activation (mediated by CD8+), as well as enhanced hydrophilic antioxidant capacity [54]. Additionally, it was reported that selenium-saturated bovine lactoferrin improved the activity of key antioxidant enzymes, such as superoxide dismutase (SOD) and catalase (CAT) at the cellular and tissue level [55]. These findings highlight the potential benefit of lactoferrin, evidencing its capacity to modulate antioxidant mechanisms and promote positive health effects.

## 5. Neuroprotective Effects Through Oxidative Stress Reduction

Oxidative stress plays a significant role in neurodegenerative diseases, making lactoferrin’s antioxidant properties particularly valuable in neuroprotection. It was demonstrated that oral administration of bovine Lf (200 mg/kg/day) in diabetic rats provided significant protection against brain tissue damage by combating oxidative stress [56]. The study highlighted that lactoferrin reduced levels of malondialdehyde (MDA), a marker of lipid peroxidation and oxidative damage while boosting the activities of antioxidant enzymes such as SOD and GPx in brain tissues. These findings suggest that lactoferrin’s neuroprotective effects are intricately linked to its ability to mitigate oxidative stress, offering potential therapeutic benefits for neurodegenerative conditions. Although lactoferrin is a protein with a high molecular weight, several findings report that this protein is detected after oral administration thanks to its ability to cross the blood-brain barrier; the neuroprotective effects of bLf are dose-dependent, with varying doses (0.1, 1, and 10 g/kg of body weight) providing early protection from 24 h up to 22 days after the hypoxic-ischemic insult. This demonstrates that the protection conferred by bLf prevents energy metabolic dysfunction and preserves the coupling between astrocytes and neurons, thereby mitigating hypoxic-ischemic damage in a dose-dependent manner [57,58].

Further supporting its neuroprotective role, [44] emphasized lactoferrin’s protective effects against oxidative stress-induced apoptosis in neural cells. By regulating iron levels and preventing the subsequent ROS generation, bovine lactoferrin reduces oxidative damage to neurons, highlighting its potential as a neuroprotective agent in conditions like Parkinson’s and Alzheimer’s diseases [59].

### 5.1. Neuroprotector Role of Lf in Parkinson’s Disease

Parkinson’s disease (PD) is the second most prevalent neurodegenerative disease after Alzheimer’s disease [60]. Among its pathological characteristics are the death of dopaminergic neurons in the black substance (SN) and a noticeable decrease in dopamine levels (DA) in the striatum [61]. Numerous studies have revealed increased iron levels in PD patients and animal models, suggesting that a dysfunction in iron metabolism in the brain is crucial to the development of PD [62]. In this context, Lf can bind to a non-heme iron and cross the blood-brain barrier to the brain via a lactoferrin-specific receptor [46]. Previous research has documented that the increase in Lf and Lf receptors in blackhead neurons suggests that Lf may play a role in iron accumulation in this region, demonstrating that Lf protects ventrally mesencephalon *neurons* in vitro by restoring mitochondrial function and modifying the ratio of Cu/Zn-SOD and expression of Bcl-2/Bax in response to 1-methyl-4-phenylpyridinium toxicity [63]. In addition, microglial activation within the substantia nigra has been implicated in the neuroinflammatory processes underlying Parkinson’s disease pathogenesis [64,65], often concomitant with excessive iron accumulation. Given that activated microglia are known to synthesize and release lactoferrin within the central nervous system, the observed increase in Lf staining within SN neurons following PD treatment suggests a potential neuroprotective mechanism [66,67]. In addition, Lf counteracted the loss of spleen weight, decreased iron particles in the spleen, and increased iron level in serum caused by MPTP toxicity, which could be associated with iron deposition in black matter [68]. Excess iron caused a decrease in the number of TH-ir neurons in the black substance due to oxidative stress and apoptosis and increased dopamine rotation in the striatum, which triggered the onset of Parkinson’s disease (PD). Therefore, Lf was shown to be a safe and effective neuroprotective agent capable of reducing brain iron [44]. Due to the broad spectrum lactoferrin has in the brain, it can also become a support molecule for therapeutic agents against PE. It can mediate receptor-mediated transcytosis, increase blood-brain barrier (BBB) permeation, and bind to DA cells that are rich in lactoferrin receptors, thus increasing specificity and ensuring the safety of the therapeutic agent pathway. The lack of data and research on the potential neuroprotection of lactoferrin against Parkinson’s disease offers a very wide field of study in the search for an alternative that could be, in the future, a safe and effective therapeutic option to manage brain anomalies and movement disorders associated with PD [69].

### 5.2. Neuroprotector Role of Lf in Alzheimer’s Disease

Alzheimer’s disease (AD) represents the main etiology of dementia and is emerging as one of the most significant, burdensome, and lethal pathologies in the course of this century [70]. By 2050, it is estimated that 1 in 85 people will suffer from the disease, and 43% of these patients will need specialized health services [71]. It is characterized by a process of neurodegeneration in vulnerable brain areas [72]. The susceptibility of the brain to reactive oxygen species (ROS) is being recognized as a key factor driving the development of AD. Oxidative stress irreversibly damages cellular biomolecules and alters neural functions. Scientific evidence supports the use of antioxidants to prevent and slow disease progression [73]. Despite the increase in the incidence of AD, current therapies fail to stop its progression, which underlines the need to investigate the pathophysiology of this disorder [74]. Recent research has shown that, in the presence of neurodegeneration, there are alterations in mitochondrial metabolic pathways, beta-amyloid peptide neurotoxicity, and alterations in the regulation of the neuronal metabolism of Ca^2+^. Although it has not been determined whether these events are the cause or consequence of the disease, they are all related to an increase in ROS production [71]. Oxidative stress is fundamental in the etiopathogenesis of AD since alterations in the electron transport chain and the production of ROS in mitochondrial respiration contribute to the development of the disease. These ROS include superoxide anion (O2-), hydrogen peroxide (H_2_O_2_), and hydroxyl radical (HO), generated by reduction reactions in the mitochondria [75]. While endogenous antioxidant systems strive to mitigate reactive oxygen species, their efficacy can be overwhelming, particularly within the highly metabolically active yet antioxidant-deficient neural environment. This inherent susceptibility to oxidative stress underscores its significance as a critical area of investigation within the context of neurodegenerative disease pathogenesis [76]. A recent study on recombinant human lactoferrin (rhLF) production using the CHO expression platform has developed a protocol for its large-scale expression and purification. rhLF showed high biological potency and compatibility with milk-derived lactoferrin, as well as having a modulating effect on the cellular redox state by positively regulating key antioxidant enzymes [77]. These results suggest that rhLF has great therapeutic potential and could overcome existing barriers to its systemic administration in humans. Lf plays a crucial role in protecting the brain, as shown by studies in patients with AD and animal models. It has been observed that human Lf is upregulated in the brains of AD patients and accumulates on senile fibrillar plaques and areas affected by amyloid angiopathy in transgenic mice APP, a model of this disease. In addition, with age, the Lf deposits in the brains of these mice increase in number and intensity, suggesting a progressive response to neurodegenerative damage. These findings indicate that lactoferrin could protect brain integrity through its anti-inflammatory and antioxidant properties, preventing cell and tissue damage [67]. In additional studies, lactoferrin levels were found to be elevated in the brains of Alzheimer’s patients, accumulating in areas with senile plaques and neurofibrillary twists. Although its exact origin in AD is uncertain, it is suggested that Lf could be locally synthesized by reactive microglia or infiltrating monocytes/macrophages, acting as a neuroprotective mechanism. It is argued that Lf from different sources (bovine and human) could inhibit the inflammatory response and reduce oxidative damage associated with AD, by suppressing inflammatory cytokines and modulating ROS and redox-active iron [38,78,79]. Lf’s ROS-modulating and anti-inflammatory effects may protect against AD by influencing the phosphorylated protein kinase B pathway (p-PKB/AKT)/phosphatase and tensin homolog (PTEN). This hypothesis has been confirmed in patients with AD. Lf can also degrade substances’ oxidative metabolism by positively regulating cytochrome P450 enzymes and other related proteins. It is proposed that Lf acts as an iron chelator, inducing the expression of HIF-1α and exerting neuroprotective effects. In addition, Lf can promote the nonamyloid metabolism of APP by activating disintegrin and metalloproteinase 10 (ADAM10), reducing the formation of Aβ plates and improving cognition and learning. These effects are related to the positive regulation of ADAM10 through the ERK1/2-CREB and HIF-1α pathways, suggesting that Lf could improve cognitive decline and serve as a protective brain response in patients with AD [38]. This indicates that rhLf may be a promising option for human therapy, overcoming barriers to its systemic administration.

## 6. Lf Antioxidant Impact on Other Systems

Oxygen, while essential for life, can contribute to disease pathogenesis through the uncontrolled production of reactive oxygen species [80]. These free radicals can induce oxidative damage to macromolecules, disrupting cellular processes and contributing to a state of oxidative stress [81]. To counteract this, organisms maintain a delicate balance between ROS production and antioxidant defense mechanisms. Oxidative stress has been implicated in the etiology and progression of over 100 diseases [82]. Evidence suggests that certain agents, such as Lf, have demonstrated the capacity to attenuate ROS levels and modulate oxidative stress responses [83].

### 6.1. Lf in Obesity

Administration of 250 mg/day of camel lactoferrin in obese pediatric patients has been shown to decrease body mass index, lipid profile, glycosylated hemoglobin, and levels of IL-1β, IL-6, IL-18, TNF-α, and lipocalin-2, and on the other hand, increase the level of superoxide dismutase (SOD), indicating its anti-inflammatory effect and antioxidant effect. This is important because obesity is often described as an inflammatory disease, and the body’s defenses are based on an enzymatic antioxidant system and endogenous and exogenous antioxidant substances [84]. Furthermore, during obesity, the progression of vascular endothelial dysfunction is promoted, as an underlying mechanism of hypertension and subsequent damage to vital organs [85]; previous studies have shown that bovine lactoferrin has antihypertensive effects through various mechanisms, such as lowering systolic blood pressure, decreasing serum adhesion molecules, and reducing reactive oxygen species levels in the aorta. An improvement in endothelium-dependent relaxation function has also been observed in mice fed a high-fat diet [86].

### 6.2. Lf in the Immunological System

During inflammation, the phagocytes release reactive oxygen species, which neutralize microorganisms; in turn, these also affect living tissue negatively, generating necrosis. At the physiological level, tissues release iron to participate in the Haber–Weiss reaction, generating more free radicals. Due to its binding to iron, lactoferrin helps relieve oxidative stress [87]. The therapeutic properties of Lf have also been seen in pathology associated with *Helicobacter pylori* and gastric injury. Recombinant human Lf has been reported to modulate the inflammatory response by interacting with surface receptors of immune cells, regulating intracellular signaling pathways and controlling the production of inflammatory cytokines and iron oxidative activity in a time- and dose-dependent action [88,89].

### 6.3. Lf in Anemia

Iron homeostasis is regulated in part by Lf because of its role in protecting against oxidative stress and reducing the amount of cell damage induced by aggression [21]. Considering this, several studies have been conducted to evaluate the efficacy of oral administration of bovine Lf in the treatment of anemia. Iron deficiency anemia is a risk factor for premature birth, so one study compared the effect of supplementation with bLf and ferrous sulfate in women in different trimesters of pregnancy; the results were that women treated with bLf had higher hemoglobin and iron levels than those who received ferrous sulfate [90]. Another study by the same authors showed that hemoglobin, serum iron, and red blood cells increased when they received bLf and decreased with ferrous sulfate [91]. In addition, decreased serum IL-6 concentrations were shown to be involved in the induction of hypoferremia and cause anemia [92]. Anemia has also been shown to be derived in endurance athletes, especially long-distance runners who menstruate and control their weight. Therefore, a study was conducted to verify whether the intake of bLf could improve or prevent this disease in athletes. The results showed that bLf increases iron absorption, suggesting its usefulness in preventing sports anemia [93].

### 6.4. Lf in Respiratory Disease

Lung diseases have become more recurrent in recent decades. In bronchial asthma, oxidative stress intensifies respiratory tract inflammation by inducing pro-inflammatory mediators, increasing bronchial hyperreactivity, and stimulating bronchospasm and mucin production [94]. It has been previously reported that the oxygen free radicals generated by nicotinamide adenine dinucleotide phosphate (NADPH) reduced oxidase from pollen grains or extracts of it, providing an improvement to allergic inflammation of the respiratory tract [95]. It was subsequently shown that bLf is able to reduce the inflammation of the respiratory tract induced by pollen extract [96]. The efficacy of hLf and bLf in reducing allergen-induced pleurisy in BALB/c mice was also studied; the results showed the effectiveness of both proteins in reducing pleurisy, generating importance in the research group since it increased knowledge of the suppressor efficacy of the protein [97]. Bovine lactoferrin has also been studied in a murine model of cystic fibrosis, known as a multifactorial disease, where iron imbalance, inflammation, and bacterial infection play an important role in the chronicity and severity of the disease. The results showed that administration in an aerosol manner was able to reduce infiltrated leukocytes and iron overload [98].

### 6.5. Lf in Hepatitis

The clinical use of Lf in animal models with an inflammatory liver has produced promising results such as the inhibition of hepatic inflammation in patients with chronic hepatitis C [99]. In an in vivo study by Tsubota et al., (2014), they used Long–Evans Cinnamon rats to develop fulminant hepatitis to evaluate the effect of bLf on oxidative liver damage, showing that the protein recovered reduced base cleavage repair capacity and also lowered accumulation levels of 8-hydroxy-20-deoxygenosine (ROS-induced DNA modification marker) and mutations in hepatic mitochondrial DNA suggesting its use as a potential treatment for oxidative stress-induced liver disease [100]. The effect of bLf on lipid peroxidation, hepatic inflammation, and iron metabolism was also evaluated in CHC patients, where an improvement in lipid peroxidation and alanine aminotransferase (ALT) levels was demonstrated, suggesting a therapeutic approach for the suppression of oxidative stress and inflammation in patients with CHC [101].

### 6.6. Lf in Dry Eyeness Disease

Topical application of bovine lactoferrin has been shown to reduce irradiation-induced corneal epithelial damage in murine models and to promote burn-out wound healing in the cornea [102]. The reason for using this protein in dry eye disease is its ability to address underlying inflammation and oxidative stress [103]. Thanks to its iron chelating activity, it provides the elimination of free radicals from oxygen and hydroxyl, inhibiting pro-inflammatory effects and harm to tissues [104]. This major protein in the tear has multiple functions, including regulation of microbial growth as well as oxidative stress [83,105]. A study made in cultured HCECs with oxidative stress induced by hydrogen peroxide, incubated with lactoferrin and xantham gum, was able to protect the cells against the effects of ROS [106,107]. Otherwise, proteins involved in the activation of host immune response, like transferrin, were found in tears of patients with Sjogren syndrome but not in patients with dry eye without Sjogren syndrome or normal volunteers [108]. Furthermore, patients with dry eye exhibited altered levels of these proteins when compared to healthy volunteers. A marked decrease in lactoferrin was identified in the dry eye group relative to the healthy participants [109]. This proteome study supports the link between dry eye and oxidative stress and suggests that the inflammation encountered in human dry eye may be associated with oxidative stress [110].

### 6.7. Lf in Cardiovascular Disease

Dexamethasone-induced hypertension (Dex) has been reported above to be associated with increased oxidative stress. In a study conducted on male Wistar rats, the effect of Lf on oxidative stress and hypertension after administration of Dex was investigated, obtaining that bovine Lf decreased and prevented dose-dependent hypertension mediated by Dex. The Lf prevented and reversed the overproduction of H_2_O_2_ during dexamethasone injection, showing significant increases in plasma iron-reducing antioxidant power after dexamethasone administration, confirming the role of oxidative stress in the pathogenesis of hypertension [41]. Its antioxidant effect on erythrocytes through inhibition of lipid peroxidation and hemolysis was also reported and has been seen to contribute to reducing oxidative reactions in the cell membrane [111].

### 6.8. Lf in Angiogenesis

Angiogenesis plays an important role in various physiological processes, such as tissue regeneration, tumor growth, metastasis, and hypoxia. VEGF-A is among the key factors promoting angiogenesis. In one study, human apo-Lf was shown to positively regulate the expression of KDR/FIk-1 (VEGF-A receptor) in human umbilical vein endothelial cells, promoting VEGF-induced proliferation and migration through phosphorylation of MAPK. On the other hand, the authors also observed that human holo-Lf did not have a significant influence even in the presence of VEGF-A [112]. In a rats’ mesenteric window angiogenesis assay study, bLf was administered to significantly inhibit VEGF165-mediated angiogenesis, achieving a spatial extension of microvessels and general vascularity [113]. Finally, a fragment of bLf (lactoferricin B) was used which could suppress the angiogenesis induced by FGF-2 as by VEGF165 in mice. It is thought that the differences observed may influence the iron release and union of the Lf [114].

## 7. Concluding Remarks

Lactoferrin stands out as a multifaceted biomolecule with remarkable potential in addressing the complex mechanisms underlying chronic diseases. Its antioxidant, anti-inflammatory, and iron-regulating properties position it as a promising therapeutic agent for conditions characterized by oxidative stress, such as cardiovascular and neurodegenerative diseases and cancer. By mitigating ROS production, modulating iron metabolism, and enhancing endogenous antioxidant defenses, Lf offers significant protection against oxidative damage and associated tissue injury. Its neuroprotective effects, particularly in Parkinson’s and Alzheimer’s diseases, underscore its relevance in neurodegeneration, where it regulates iron accumulation, combats inflammation, and prevents neuronal apoptosis. Furthermore, Lf’s ability to support systemic homeostasis, including its roles in immune modulation and antimicrobial activity, broadens its therapeutic applications. Despite these promising findings, further research is needed to translate these insights into clinical applications, optimizing its delivery, dosage, and long-term safety. Ultimately, Lf represents a valuable avenue for developing novel, effective strategies to combat oxidative stress-driven pathologies, potentially improving outcomes for millions worldwide.
